# Crystal structure of a dimeric β-diketiminate magnesium complex

**DOI:** 10.1107/S2056989016017394

**Published:** 2016-11-04

**Authors:** Connor S. MacNeil, Kevin R. D. Johnson, Paul G. Hayes, René T. Boeré

**Affiliations:** a Department of Chemistry and Biochemistry, University of Lethbridge, Lethbridge, AB, T1K 3M4, Canada

**Keywords:** single-crystal X-ray study, low oxidation, β-diketiminates, dimeric magnesium complex

## Abstract

The solid-state structure of a dimeric β-diketiminate magnesium(II) complex is discussed. The compound, di-μ-iodido-bis­[(­{4-amino-1,5-bis­[2,6-bis­(propan-2-yl)phen­yl]pent-3-en-2-yl­idene}aza­nido-κ^2^ *N*,*N*′)magnesium(II)] toluene sesquisolvate, [Mg_2_(C_29_H_41_N_2_)_2_I_2_]·1.5C_7_H_8_, crystallizes as two independent mol­ecules, each with 2/*m* crystallographic site symmetry, located at Wyckoff sites 2*c* and 2*d*. These have symmetry-equivalent magnesium atoms bridged by μ-iodide ligands with very similar Mg—I distances. The two Mg atoms are located slightly below (∼0.5 Å) the least-squares plane defined by N–C—C–N atoms in the ligand scaffold, and are approximately tetra­hedrally coordinated. One and one-half toluene solvent mol­ecules are disordered with respect to mirror-site symmetry at Wyckoff sites 4*i* and 2*a*, respectively. In the former case, two toluene mol­ecules inter­act in an off-center parallel stacking arrangement; the shortest C to C′ (π–π) distance of 3.72 (1) Å was measured for this inter­action.

## Abstract 2

The crystal structure of a dimeric β-diketiminate magnesium(II) complex crystallizes as two independent mol­ecules, each with 2/*m* crystallographic site symmetry, located at Wyckoff sites 2*c* and 2*d*. These have symmetry-equivalent magnesium atoms bridged by μ-iodide ligands with very similar Mg—I distances.

## Chemical context   

The ubiquity of β-diketiminate (Nacnac) ancillary ligands likely stems from the analogous acetyl­acetonato (acac) ligands in coordination chemistry. This nitro­gen analogue of acac allows for modular electronic and steric tuning of the ligand framework by altering groups on the N atoms, which engenders marked stability in low-oxidation-state metals as described in the literature, notably by the late Professor Lappert (Bourget-Merle *et al.*, 2002 ▸). Whereas the chemistry of group 2 metals is largely defined by the +2 oxidation state, a landmark contribition by Cameron Jones and co-workers, describing an Mg^I^ complex bearing a Nacnac ligand, and containing a covalent magnesium–magnesium bond [Mg—Mg = 2.8457 (8) Å] was reported (Green *et al.*, 2007 ▸). Important precursors in the synthesis of these Mg^II^ compounds are dimeric hydride- and iodide-bridged Mg^II^I complexes bearing Nacnac ligands of varying steric bulk. Notably, only one other β-diketiminate-stabilized Mg_2_I_2_ complex has been characterized crystallographically (Bonyhady *et al.*, 2010 ▸). This report describes a previously unreported Mg_2_I_2_ complex which crystallizes as two independent dimers, suitable for direct comparison of metrical data.

## Structural commentary   

**Figure 1:**
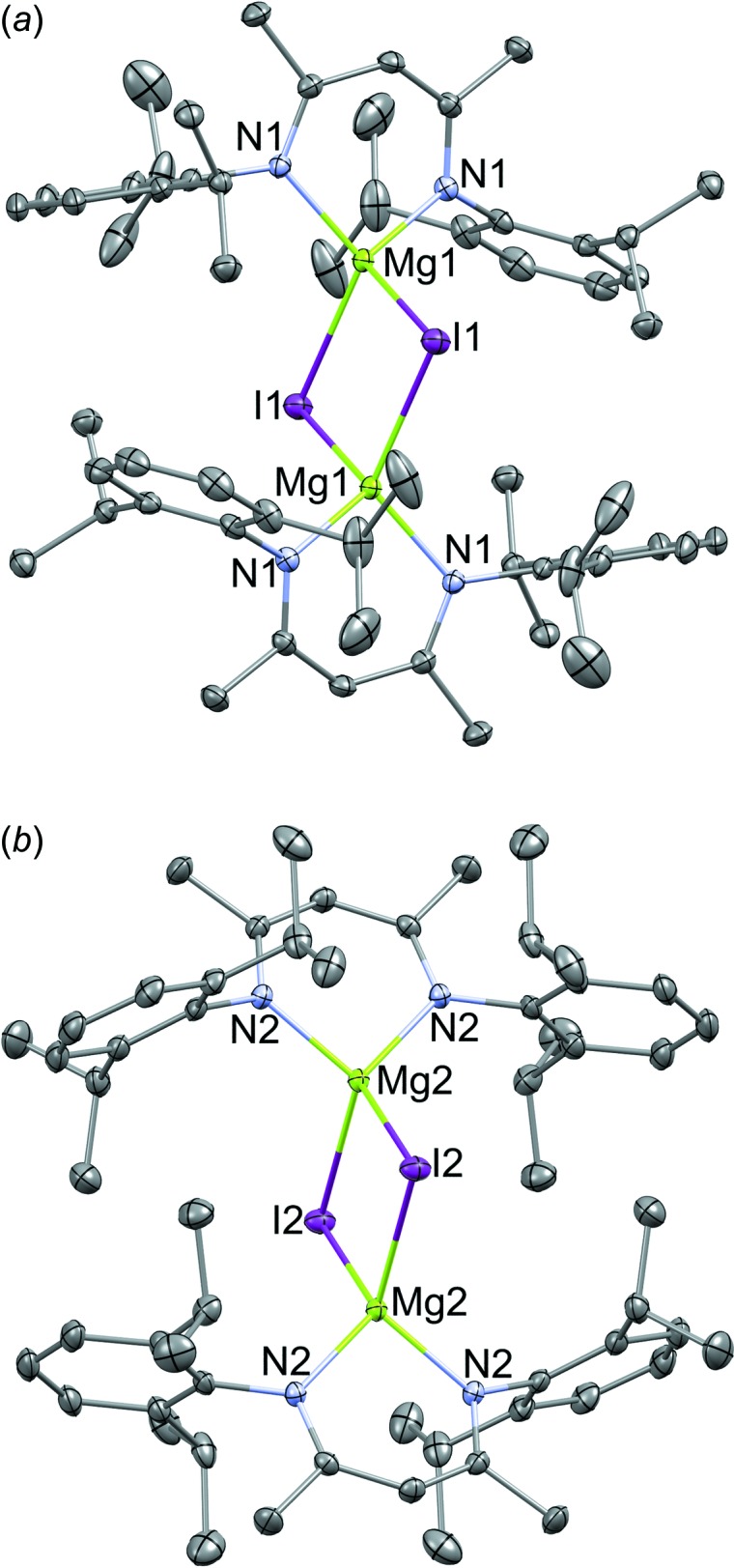
Displacement ellipsoids diagram (50% probability level) of the molecular structure of the title compound, showing the two independent metal environments for Mg1 (*a*) and Mg2 (*b*), each with site symmetry of 2/*m*. Each metal is associated with one iodine and half a Nacnac ligand that are crystallographically independent. Hydrogen atoms and disordered toluene mol­ecules have been omitted for clarity.

The title compound (Fig. 1 ▸) is reported as crystallographically independent dimers in the monoclinic space group *C*2/*m*. Each dimer has 2/*m* symmetry, thus only ¼ of each mol­ecule is unique. 
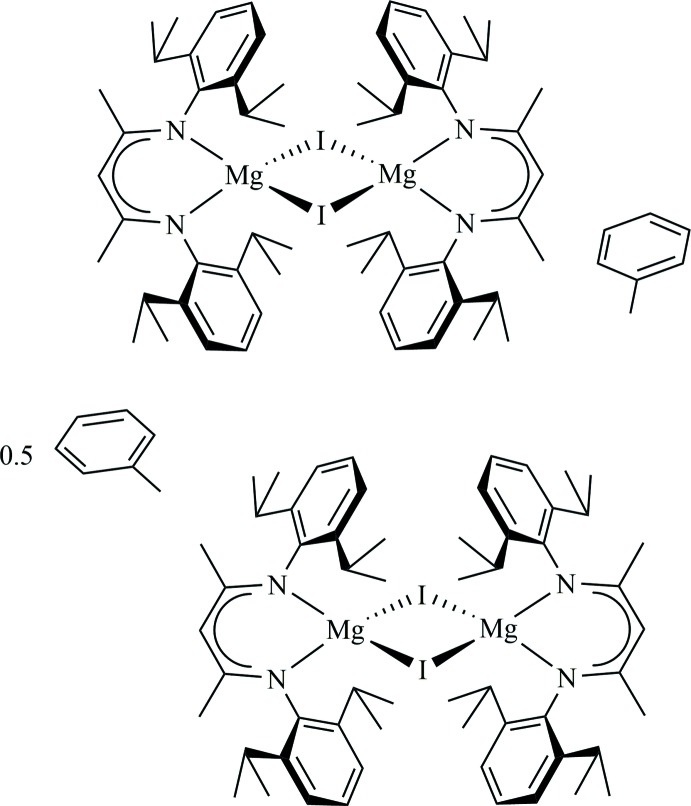


Both Mg_2_I_2_ fragments are crystallographically orthogonal to the coordinating Nacnac ligand scaffolds. The magnesium atoms are located 0.512 (4) Å and 0.473 (3) Å out of the mean least-square plane defined by (N1–C1–C1–N1) or (N2–C21–C21–N2), which suggests predominantly κ^2^ bonding. Chelate-ring atoms C2 and C22 deviate from these planes by 0.127 (4) and 0.111 (4) Å, respectively. The coordination geometry is best described as stated; however, there are reports of low-lying *p* orbitals contributing to more pronounced deviations of the metal from the calculated plane of the ligand (Randall *et al.*, 2000 ▸). The geometry about each magnesium atom is pseudo-tetra­hedral; the average tetra­hedral angle is 106.6°. The backbone of the ligand is not strictly planar; identical torsion angles were measured [Mg1—N1—C1—C2 = 10.2 (3) and Mg2—N2—C21—C22 = 10.1 (3)°]. Mg—I bond lengths [Mg1—I1 = 2.7718 (9) Å, Mg2—I2 = 2.7581 (9) Å] are comparable to those previously reported in a similar structure [Mg1—I1 = 2.7471 (10), Mg1—I1′ = 2.7667 (11) Å; Bonyhady *et al.*, 2010 ▸]. Likewise, the Mg—I—Mg’ angles compare well with the previously reported structure, and are equal within error in the present crystal [Mg1—I1—Mg1′ = 83.62 (3), I1—Mg1—I1′ = 96.38 (3)° and Mg2—I2—Mg2′ = 83.14 (3), I2—Mg2—I2′ = 96.86 (3)°].

## Synthesis and crystallization   

Under a dry, argon atmosphere, an oven-dried Schlenk flask was charged with lithium ^Dip^Nacnac (3.71 g, 8.74 mmol), MgI_2_ (2.45 g, 8.80 mmol), and a magnetic stir bar. Diethyl ether (70 mL, dried over sodium benzo­phenone ket­yl) was condensed into the flask at 195 K by vacuum transfer, providing a cloudy beige solution. The reaction mixture was warmed to 273 K and stirred for three h. The flask was then warmed to room temperature and all volatiles were removed under reduced pressure. The resulting white residue was reconstituted in toluene and passed through a fine porosity frit, affording a clear-yellow filtrate. The filtrate was concentrated under vacuum and single crystals suitable for diffraction were grown from this concentrated toluene solution at 238 K. ^1^H NMR (CDCl_3_): δ 7.12 (*t*, ^3^ *J*_HH_ = 7.6 Hz, 4H, aromatic C*H*), 6.97 (*d*, ^3^ *J*_HH_ = 7.6 Hz, 8H, aromatic C*H*), 4.76 (*s*, 2H, NC(CH_3_)C*H*), 3.02 (*sp*, ^3^ *J*_HH_ = 6.8 Hz, 8H, C*H*(CH_3_)_2_), 1.57 (*s*, 12H, NC(C*H*_3_)CH), 1.04 [*d*, ^3^ *J*_HH_ = 6.8 Hz, 24H, CH(C*H*_3_)(CH_3_)], 0.78 [*d*, ^3^ *J*_HH_ = 6.8 Hz, 24H, CH(CH_3_)(C*H*_3_)]. ^13^C{^1^H} NMR (CDCl_3_): δ 169.9 (N*C*(CH_3_)CH), 143.6 (aromatic *C*), 142.6 (aromatic *C*), 125.6 (aromatic *C*H), 123.8 (aromatic *C*H), 94.8 (NC(CH_3_)*C*H), 28.0 (*C*H(CH_3_)_2_), 25.6 (CH(CH_3_)(*C*H_3_)), 24.7 (CH(*C*H_3_)(CH_3_)), 24.4 (NC(*C*H_3_)CH). Analysis calculated for C_58_H_82_I_2_Mg_2_N_4_: C, 61.23; H, 7.26; N, 4.92. Found: C, 61.06; H, 6.98; N, 5.14.

## Refinement   

In the crystal, toluene mol­ecules occupy Wyckoff special sites 2*a* and 4*i* and are disordered w.r.t. the 2/*m* and *m* symmetry, respectively; surprisingly both lie with their mol­ecular planes *perpendicular* to the crystallographic mirror. Both toluene mol­ecules are (at least approximately) coplanar with the typical orientations where the methyl carbons C47 and C57 lie close to C44 and C54 of the rings, consistent with inter­actions of aromatic solvents (Martinez & Iverson, 2012 ▸). These disordered groups also have large displacement parameters indicative of considerable freedom of motion within the solvent cavities. Anisotropic refinement proceeded after applying similarity, ring flatness and approximate isotropic displacement restraints on all the solvent carbon atoms (with s.u. of 0.1 on each restraint). In addition the C41–C47 distance was constrained to 1.45±0.01 Å and the displacement ellipsoids of C54 and C57 were constrained to be the same. A more technical description is provided in the CIF file and an archival RES file has been provided. Crystal data, data collection and structure refinement details are summarized in Table 1 ▸.

## Supplementary Material

Crystal structure: contains datablock(s) I. DOI: 10.1107/S2056989016017394/zl2683sup1.cif

Structure factors: contains datablock(s) I. DOI: 10.1107/S2056989016017394/zl2683Isup2.hkl

CCDC reference: 1512965

Additional supporting information: crystallographic information; 3D view; checkCIF report

## Figures and Tables

**Table 1:** Experimental details

Crystal data	
Chemical formula	[Mg_2_(C_29_H_41_N_2_)_2_I_2_]·1.5C_7_H_8_
*M*_r_	1275.89
Crystal system, space group	Monoclinic, *C*2/*m*
Temperature (K)	173
*a*, *b*, *c* (Å)	19.1596 (15), 21.0532 (16), 16.5711 (13)
β (°)	99.9350 (8)
*V* (Å^3^)	6584.1 (9)
*Z*	4
Radiation type	Mo *K*α
μ (mm^−1^)	1.02
Crystal size (mm)	0.30 × 0.25 × 0.05

Data collection	
Diffractometer	Bruker APEXII CCD area-detector
Absorption correction	Multi-scan (*SADABS*; Bruker, 2008 ▸)
*T*_min_, *T*_max_	0.685, 0.746
No. of measured, independent and observed [*I* > 2σ(*I*)] reflections	40851, 8630, 7569
*R*_int_	0.039
(sin θ/λ)_max_ (Å^−1^)	0.687

Refinement	
*R*[*F* ^2^ > 2σ(*F* ^2^)], *wR*(*F* ^2^), *S*	0.029, 0.087, 1.06
No. of reflections	8630
No. of parameters	425
No. of restraints	150
H-atom treatment	H-atom parameters constrained
Δρ_max_, Δρ_min_ (e Å^−3^)	1.10, −0.85

Computer programs: *APEX2* and *SAINT-Plus* (Bruker, 2008 ▸), *SHELXT* (Sheldrick, 2015*a* ▸), *SHELXL2014* (Sheldrick, 2015*b* ▸), *DIAMOND* (Brandenburg, 1999 ▸) and *publCIF* (Westrip, 2010 ▸).

## supplementary crystallographic information

### Crystal data

**Table:**

[Mg_2_(C_29_H_41_N_2_)_2_I_2_]·1.5C_7_H_8_	*F*(000) = 2652
*M**_r_* = 1275.89	*D*_x_ = 1.287 Mg m^−^^3^
Monoclinic, *C*2/*m*	Mo *K*α radiation, λ = 0.71073 Å
*a* = 19.1596 (15) Å	Cell parameters from 9807 reflections
*b* = 21.0532 (16) Å	θ = 2.2–29.0°
*c* = 16.5711 (13) Å	µ = 1.02 mm^−^^1^
β = 99.9350 (8)°	*T* = 173 K
*V* = 6584.1 (9) Å^3^	Plate, colourless
*Z* = 4	0.3 × 0.25 × 0.05 mm

### Data collection

**Table:**

Bruker APEXII CCD area-detector diffractometer	8630 independent reflections
Radiation source: fine-focus sealed tube, Bruker D8	7569 reflections with *I* > 2σ(*I*)
Graphite monochromator	*R*_int_ = 0.039
Detector resolution: 66.06 pixels mm^-1^	θ_max_ = 29.2°, θ_min_ = 1.5°
φ and ω scans	*h* = −26→25
Absorption correction: multi-scan (SADABS; Bruker, 2008)	*k* = −28→28
*T*_min_ = 0.685, *T*_max_ = 0.746	*l* = −21→22
40851 measured reflections	

### Refinement

**Table:**

Refinement on *F*^2^	Primary atom site location: dual
Least-squares matrix: full	Hydrogen site location: inferred from neighbouring sites
*R*[*F*^2^ > 2σ(*F*^2^)] = 0.029	H-atom parameters constrained
*wR*(*F*^2^) = 0.087	*w* = 1/[σ^2^(*F*_o_^2^) + (0.047*P*)^2^ + 7.993*P*] where *P* = (*F*_o_^2^ + 2*F*_c_^2^)/3
*S* = 1.06	(Δ/σ)_max_ = 0.001
8630 reflections	Δρ_max_ = 1.10 e Å^−^^3^
425 parameters	Δρ_min_ = −0.85 e Å^−^^3^
150 restraints	

### Special details

**Table:**

Experimental. A crystal coated in Paratone (TM) oil was mounted on the end of a fine glass capillary and cooled in the gas stream of the diffractometer Kryoflex device.
Geometry. All esds (except the esd in the dihedral angle between two l.s. planes) are estimated using the full covariance matrix. The cell esds are taken into account individually in the estimation of esds in distances, angles and torsion angles; correlations between esds in cell parameters are only used when they are defined by crystal symmetry. An approximate (isotropic) treatment of cell esds is used for estimating esds involving l.s. planes.
Refinement. During refinement, peaks corresponding to about two toluene solvent molecules were found in two different locations in the lattice. The first is disposed about a site of mirror symmetry, whilst the second has 2/m symmetry. Both have been sucessfully modelled as a head-to-tail two-part disorders. Both were treated using the PART-1 instruction in SHELXL. The correctness of the model was checked by Platon 'squeeze' calculation (with the toluene molecules removed from the model). This confirmed the two cavities at (0 0 0) holding one solvent and at (0.5 0 0.5) holding two toluenes. The total electron count was 282 in excellent agreement with the model. The solvent disorder model is therefore considered to be acceptable and the observed large displacement elipsoids for solvent accord with the large cavities that they occupy [note that two toluene molecules occupy the large cavity (vol 420 A^3^) at (0.5 0 0.5) and are related centrosymmetrically]. In view of the satisfactory disorder model, the 'squeezed' alternative has not pursued further.

### Fractional atomic coordinates and isotropic or equivalent isotropic displacement parameters (Å^2^)

**Table:**

	*x*	*y*	*z*	*U*_iso_*/*U*_eq_	Occ. (<1)
I1	0.46274 (2)	0.5000	0.37517 (2)	0.03058 (6)	
Mg1	0.59211 (4)	0.5000	0.48017 (5)	0.02150 (16)	
N1	0.65995 (8)	0.57257 (7)	0.47110 (9)	0.0224 (3)	
C1	0.72901 (10)	0.56076 (9)	0.49351 (11)	0.0257 (4)	
C2	0.75816 (14)	0.5000	0.51065 (17)	0.0284 (5)	
H2	0.8058	0.5000	0.5387	0.034*	
C3	0.78216 (11)	0.61457 (10)	0.50034 (14)	0.0358 (4)	
H3A	0.7672	0.6454	0.4564	0.054*	
H3B	0.8289	0.5976	0.4954	0.054*	
H3C	0.7848	0.6355	0.5536	0.054*	
C4	0.63813 (10)	0.63530 (8)	0.44142 (11)	0.0253 (3)	
C5	0.62373 (12)	0.64509 (10)	0.35578 (12)	0.0355 (4)	
C6	0.60249 (12)	0.70552 (11)	0.32644 (14)	0.0410 (5)	
H6	0.5930	0.7128	0.2690	0.049*	
C7	0.59498 (11)	0.75462 (10)	0.37861 (14)	0.0392 (5)	
H7	0.5809	0.7954	0.3573	0.047*	
C8	0.60787 (11)	0.74456 (9)	0.46182 (13)	0.0340 (4)	
H8	0.6020	0.7787	0.4975	0.041*	
C9	0.62950 (9)	0.68511 (9)	0.49528 (12)	0.0264 (4)	
C10	0.63386 (19)	0.59313 (12)	0.29663 (14)	0.0576 (8)	
H10	0.6325	0.5517	0.3258	0.069*	
C11	0.7059 (2)	0.5988 (2)	0.27163 (19)	0.0821 (11)	
H11A	0.7110	0.6412	0.2490	0.123*	
H11B	0.7104	0.5666	0.2301	0.123*	
H11C	0.7429	0.5922	0.3196	0.123*	
C12	0.5753 (2)	0.59180 (17)	0.22101 (17)	0.0808 (12)	
H12A	0.5293	0.5861	0.2382	0.121*	
H12B	0.5838	0.5565	0.1854	0.121*	
H12C	0.5755	0.6319	0.1910	0.121*	
C13	0.64282 (10)	0.67773 (9)	0.58785 (12)	0.0275 (4)	
H13	0.6631	0.6345	0.6017	0.033*	
C14	0.69583 (12)	0.72759 (10)	0.62973 (13)	0.0361 (4)	
H14A	0.7395	0.7255	0.6065	0.054*	
H14B	0.7068	0.7189	0.6887	0.054*	
H14C	0.6750	0.7701	0.6207	0.054*	
C15	0.57359 (12)	0.68351 (11)	0.62128 (14)	0.0383 (5)	
H15A	0.5547	0.7266	0.6115	0.058*	
H15B	0.5825	0.6749	0.6803	0.058*	
H15C	0.5392	0.6528	0.5935	0.058*	
I2	0.51634 (2)	1.0000	0.12648 (2)	0.03119 (6)	
Mg2	0.59599 (4)	1.0000	0.00282 (5)	0.02144 (16)	
N2	0.66642 (8)	0.92871 (7)	0.00180 (9)	0.0225 (3)	
C21	0.72597 (10)	0.93959 (9)	−0.02728 (11)	0.0253 (3)	
C22	0.74947 (14)	1.0000	−0.04657 (16)	0.0272 (5)	
H22	0.7870	1.0000	−0.0774	0.033*	
C23	0.77445 (12)	0.88511 (10)	−0.03973 (15)	0.0384 (5)	
H23A	0.7525	0.8597	−0.0869	0.058*	
H23B	0.7824	0.8584	0.0094	0.058*	
H23C	0.8199	0.9019	−0.0498	0.058*	
C24	0.65529 (10)	0.86588 (8)	0.03156 (11)	0.0250 (3)	
C25	0.62506 (10)	0.81781 (9)	−0.02154 (13)	0.0297 (4)	
C26	0.61649 (12)	0.75767 (9)	0.01142 (15)	0.0377 (5)	
H26	0.5963	0.7244	−0.0237	0.045*	
C27	0.63679 (13)	0.74582 (10)	0.09373 (15)	0.0409 (5)	
H27	0.6315	0.7044	0.1145	0.049*	
C28	0.66474 (12)	0.79370 (10)	0.14621 (14)	0.0377 (5)	
H28	0.6778	0.7851	0.2031	0.045*	
C29	0.67400 (11)	0.85467 (9)	0.11669 (12)	0.0303 (4)	
C30	0.60077 (12)	0.82777 (10)	−0.11274 (13)	0.0347 (4)	
H30	0.6122	0.8724	−0.1263	0.042*	
C31	0.52111 (13)	0.81844 (13)	−0.13632 (16)	0.0476 (6)	
H31A	0.4968	0.8450	−0.1013	0.071*	
H31B	0.5094	0.7737	−0.1290	0.071*	
H31C	0.5059	0.8306	−0.1938	0.071*	
C32	0.63876 (16)	0.78248 (14)	−0.16440 (17)	0.0569 (7)	
H32A	0.6263	0.7939	−0.2224	0.085*	
H32B	0.6240	0.7387	−0.1566	0.085*	
H32C	0.6901	0.7861	−0.1469	0.085*	
C33	0.70560 (13)	0.90637 (10)	0.17521 (13)	0.0397 (5)	
H33	0.6898	0.9480	0.1493	0.048*	
C34	0.67992 (17)	0.90348 (13)	0.25765 (15)	0.0532 (7)	
H34A	0.6978	0.8647	0.2868	0.080*	
H34B	0.6280	0.9033	0.2483	0.080*	
H34C	0.6975	0.9406	0.2906	0.080*	
C35	0.78677 (15)	0.90533 (15)	0.18808 (17)	0.0580 (7)	
H35A	0.8025	0.9141	0.1360	0.087*	
H35B	0.8040	0.8634	0.2082	0.087*	
H35C	0.8058	0.9378	0.2283	0.087*	
C41	0.9540 (5)	0.4720 (3)	0.3549 (5)	0.102 (3)	0.5
C42	1.0143 (4)	0.5045 (4)	0.3919 (5)	0.187 (5)	0.5
H42	1.0542	0.4816	0.4192	0.225*	0.5
C43	1.0160 (4)	0.5704 (4)	0.3890 (5)	0.152 (5)	0.5
H43	1.0572	0.5926	0.4143	0.182*	0.5
C44	0.9576 (5)	0.6039 (3)	0.3491 (6)	0.121 (5)	0.5
H44	0.9588	0.6490	0.3471	0.145*	0.5
C45	0.8974 (4)	0.5714 (5)	0.3120 (5)	0.126 (4)	0.5
H45	0.8574	0.5942	0.2848	0.151*	0.5
C46	0.8956 (3)	0.5054 (5)	0.3149 (4)	0.144 (3)	0.5
H46	0.8544	0.4832	0.2896	0.173*	0.5
C47	0.9551 (10)	0.4064 (6)	0.3783 (11)	0.182 (8)	0.5
H47A	0.9814	0.3818	0.3432	0.273*	0.5
H47B	0.9065	0.3904	0.3721	0.273*	0.5
H47C	0.9783	0.4022	0.4356	0.273*	0.5
C51	0.4909 (5)	0.5329 (5)	0.0100 (9)	0.062 (3)	0.25
C52	0.4451 (7)	0.5090 (8)	0.0664 (8)	0.089 (4)	0.25
H52	0.4180	0.5332	0.0983	0.107*	0.25
C53	0.4504 (9)	0.4432 (8)	0.0627 (11)	0.096 (4)	0.25
H53	0.4248	0.4181	0.0953	0.115*	0.25
C54	0.4909 (5)	0.4132 (9)	0.0138 (13)	0.094 (3)	0.25
H54	0.4863	0.3683	0.0150	0.113*	0.25
C55	0.5385 (7)	0.4335 (7)	−0.0385 (9)	0.065 (3)	0.25
H55	0.5662	0.4074	−0.0675	0.078*	0.25
C56	0.5355 (5)	0.5010 (10)	−0.0379 (6)	0.060 (2)	0.25
H56	0.5632	0.5249	−0.0694	0.072*	0.25
C57	0.4909 (5)	0.6025 (7)	0.0146 (16)	0.094 (3)	0.25
H57A	0.5397	0.6182	0.0203	0.141*	0.25
H57B	0.4704	0.6160	0.0621	0.141*	0.25
H57C	0.4627	0.6199	−0.0355	0.141*	0.25

### Atomic displacement parameters (Å^2^)

**Table:**

	*U*^11^	*U*^22^	*U*^33^	*U*^12^	*U*^13^	*U*^23^
I1	0.02707 (10)	0.04085 (11)	0.02362 (9)	0.000	0.00375 (6)	0.000
Mg1	0.0218 (4)	0.0190 (4)	0.0237 (4)	0.000	0.0038 (3)	0.000
N1	0.0245 (7)	0.0217 (7)	0.0208 (7)	−0.0015 (5)	0.0030 (5)	0.0019 (5)
C1	0.0250 (8)	0.0290 (9)	0.0231 (8)	−0.0044 (7)	0.0044 (7)	−0.0005 (7)
C2	0.0193 (11)	0.0334 (14)	0.0308 (13)	0.000	−0.0001 (10)	0.000
C3	0.0269 (9)	0.0334 (10)	0.0468 (12)	−0.0079 (8)	0.0060 (8)	−0.0005 (9)
C4	0.0250 (8)	0.0240 (8)	0.0257 (8)	−0.0053 (7)	0.0012 (7)	0.0060 (7)
C5	0.0450 (12)	0.0328 (10)	0.0274 (9)	−0.0100 (9)	0.0021 (8)	0.0079 (8)
C6	0.0444 (12)	0.0425 (12)	0.0325 (10)	−0.0090 (10)	−0.0031 (9)	0.0178 (9)
C7	0.0333 (10)	0.0321 (10)	0.0489 (13)	−0.0017 (8)	−0.0021 (9)	0.0189 (9)
C8	0.0300 (10)	0.0258 (9)	0.0444 (11)	0.0017 (7)	0.0015 (8)	0.0078 (8)
C9	0.0219 (8)	0.0238 (8)	0.0323 (9)	−0.0010 (6)	0.0010 (7)	0.0054 (7)
C10	0.111 (2)	0.0386 (12)	0.0219 (10)	−0.0106 (14)	0.0087 (12)	0.0047 (9)
C11	0.103 (3)	0.104 (3)	0.0386 (15)	0.033 (2)	0.0097 (16)	−0.0168 (16)
C12	0.131 (3)	0.077 (2)	0.0299 (13)	−0.055 (2)	0.0009 (16)	0.0018 (13)
C13	0.0289 (9)	0.0227 (8)	0.0301 (9)	0.0018 (7)	0.0031 (7)	0.0005 (7)
C14	0.0353 (11)	0.0335 (10)	0.0365 (11)	−0.0024 (8)	−0.0024 (8)	−0.0025 (8)
C15	0.0336 (10)	0.0384 (11)	0.0448 (12)	−0.0001 (9)	0.0120 (9)	−0.0006 (9)
I2	0.02600 (10)	0.04328 (11)	0.02399 (9)	0.000	0.00348 (6)	0.000
Mg2	0.0217 (4)	0.0177 (4)	0.0251 (4)	0.000	0.0045 (3)	0.000
N2	0.0246 (7)	0.0191 (7)	0.0239 (7)	0.0018 (5)	0.0046 (6)	0.0003 (5)
C21	0.0259 (8)	0.0256 (8)	0.0249 (8)	0.0041 (7)	0.0053 (7)	−0.0017 (7)
C22	0.0268 (12)	0.0301 (13)	0.0270 (12)	0.000	0.0110 (10)	0.000
C23	0.0348 (11)	0.0321 (10)	0.0521 (13)	0.0071 (8)	0.0181 (9)	−0.0028 (9)
C24	0.0255 (8)	0.0183 (8)	0.0325 (9)	0.0047 (6)	0.0083 (7)	0.0017 (7)
C25	0.0301 (9)	0.0231 (8)	0.0378 (10)	0.0021 (7)	0.0117 (8)	−0.0028 (7)
C26	0.0415 (11)	0.0208 (9)	0.0536 (13)	−0.0003 (8)	0.0158 (10)	−0.0038 (8)
C27	0.0461 (12)	0.0221 (9)	0.0580 (14)	0.0059 (8)	0.0184 (11)	0.0093 (9)
C28	0.0425 (12)	0.0298 (10)	0.0418 (11)	0.0093 (9)	0.0096 (9)	0.0125 (9)
C29	0.0322 (10)	0.0243 (9)	0.0339 (10)	0.0058 (7)	0.0048 (8)	0.0049 (7)
C30	0.0392 (11)	0.0309 (10)	0.0351 (10)	−0.0042 (8)	0.0096 (8)	−0.0097 (8)
C31	0.0404 (12)	0.0484 (13)	0.0522 (14)	−0.0062 (10)	0.0029 (11)	−0.0091 (11)
C32	0.0612 (17)	0.0616 (17)	0.0517 (15)	0.0024 (13)	0.0203 (13)	−0.0234 (13)
C33	0.0564 (13)	0.0305 (10)	0.0279 (10)	0.0045 (9)	−0.0047 (9)	0.0059 (8)
C34	0.0751 (19)	0.0495 (14)	0.0329 (12)	0.0168 (13)	0.0028 (12)	0.0024 (10)
C35	0.0578 (16)	0.0669 (18)	0.0445 (14)	−0.0163 (14)	−0.0046 (12)	0.0016 (12)
C41	0.115 (6)	0.089 (5)	0.118 (6)	0.009 (5)	0.062 (5)	−0.015 (4)
C42	0.155 (7)	0.176 (8)	0.213 (8)	0.027 (10)	−0.018 (7)	0.015 (10)
C43	0.136 (8)	0.139 (8)	0.163 (8)	−0.019 (7)	−0.022 (7)	0.026 (7)
C44	0.151 (9)	0.084 (6)	0.136 (8)	0.002 (6)	0.051 (7)	0.035 (6)
C45	0.092 (6)	0.149 (8)	0.143 (8)	0.001 (6)	0.042 (6)	0.035 (6)
C46	0.104 (5)	0.161 (7)	0.176 (7)	−0.022 (9)	0.048 (5)	−0.012 (9)
C47	0.231 (16)	0.135 (11)	0.221 (15)	−0.011 (11)	0.150 (13)	−0.034 (11)
C51	0.051 (6)	0.070 (5)	0.054 (6)	0.014 (5)	−0.019 (4)	−0.011 (5)
C52	0.071 (6)	0.085 (7)	0.095 (6)	0.007 (5)	−0.032 (5)	−0.006 (6)
C53	0.072 (6)	0.112 (7)	0.093 (7)	−0.002 (6)	−0.017 (5)	0.001 (6)
C54	0.078 (5)	0.107 (6)	0.084 (6)	0.001 (5)	−0.022 (4)	0.001 (5)
C55	0.047 (5)	0.081 (6)	0.059 (5)	−0.015 (5)	−0.011 (4)	0.016 (5)
C56	0.049 (4)	0.077 (5)	0.046 (4)	0.000 (6)	−0.010 (3)	0.001 (6)
C57	0.078 (5)	0.107 (6)	0.084 (6)	0.001 (5)	−0.022 (4)	0.001 (5)

### Geometric parameters (Å, º)

**Table:**

I1—Mg1	2.7718 (9)	C24—C29	1.414 (3)
I1—Mg1^i^	2.7785 (9)	C25—C26	1.400 (3)
Mg1—I1^i^	2.7785 (9)	C25—C30	1.517 (3)
Mg1—N1^ii^	2.0283 (16)	C26—H26	0.9500
Mg1—N1	2.0283 (16)	C26—C27	1.375 (3)
N1—C1	1.335 (2)	C27—H27	0.9500
N1—C4	1.446 (2)	C27—C28	1.378 (3)
C1—C2	1.405 (2)	C28—H28	0.9500
C1—C3	1.514 (3)	C28—C29	1.396 (3)
C2—C1^ii^	1.405 (2)	C29—C33	1.513 (3)
C2—H2	0.9500	C30—H30	1.0000
C3—H3A	0.9800	C30—C31	1.522 (3)
C3—H3B	0.9800	C30—C32	1.545 (3)
C3—H3C	0.9800	C31—H31A	0.9800
C4—C5	1.413 (3)	C31—H31B	0.9800
C4—C9	1.405 (3)	C31—H31C	0.9800
C5—C6	1.397 (3)	C32—H32A	0.9800
C5—C10	1.504 (3)	C32—H32B	0.9800
C6—H6	0.9500	C32—H32C	0.9800
C6—C7	1.371 (4)	C33—H33	1.0000
C7—H7	0.9500	C33—C34	1.531 (3)
C7—C8	1.375 (3)	C33—C35	1.533 (4)
C8—H8	0.9500	C34—H34A	0.9800
C8—C9	1.402 (3)	C34—H34B	0.9800
C9—C13	1.519 (3)	C34—H34C	0.9800
C10—H10	1.0000	C35—H35A	0.9800
C10—C11	1.513 (5)	C35—H35B	0.9800
C10—C12	1.531 (4)	C35—H35C	0.9800
C11—H11A	0.9800	C41—C42	1.3900
C11—H11B	0.9800	C41—C46	1.3900
C11—H11C	0.9800	C41—C47	1.433 (8)
C12—H12A	0.9800	C42—H42	0.9500
C12—H12B	0.9800	C42—C43	1.3900
C12—H12C	0.9800	C43—H43	0.9500
C13—H13	1.0000	C43—C44	1.3900
C13—C14	1.540 (3)	C44—H44	0.9500
C13—C15	1.528 (3)	C44—C45	1.3900
C14—H14A	0.9800	C45—H45	0.9500
C14—H14B	0.9800	C45—C46	1.3900
C14—H14C	0.9800	C46—H46	0.9500
C15—H15A	0.9800	C47—H47A	0.9800
C15—H15B	0.9800	C47—H47B	0.9800
C15—H15C	0.9800	C47—H47C	0.9800
I2—Mg2	2.7581 (9)	C51—C52	1.476 (14)
I2—Mg2^iii^	2.7627 (9)	C51—C56	1.430 (13)
Mg2—I2^iii^	2.7627 (9)	C51—C57	1.468 (15)
Mg2—Mg2^iii^	3.6634 (17)	C52—H52	0.9500
Mg2—N2^iv^	2.0203 (16)	C52—C53	1.391 (17)
Mg2—N2	2.0203 (16)	C53—H53	0.9500
N2—C21	1.333 (2)	C53—C54	1.370 (16)
N2—C24	1.440 (2)	C54—H54	0.9500
C21—C22	1.404 (2)	C54—C55	1.427 (16)
C21—C23	1.512 (3)	C55—H55	0.9500
C22—C21^iv^	1.404 (2)	C55—C56	1.423 (18)
C22—H22	0.9500	C56—H56	0.9500
C23—H23A	0.9800	C57—H57A	0.9800
C23—H23B	0.9800	C57—H57B	0.9800
C23—H23C	0.9800	C57—H57C	0.9800
C24—C25	1.399 (3)		
			
Mg1—I1—Mg1^i^	83.62 (3)	C25—C24—N2	121.24 (16)
I1—Mg1—I1^i^	96.38 (3)	C25—C24—C29	120.85 (17)
N1^ii^—Mg1—I1	117.94 (5)	C29—C24—N2	117.89 (16)
N1—Mg1—I1	117.94 (5)	C24—C25—C26	118.20 (19)
N1^ii^—Mg1—I1^i^	113.99 (5)	C24—C25—C30	123.29 (17)
N1—Mg1—I1^i^	113.99 (5)	C26—C25—C30	118.51 (18)
N1—Mg1—N1^ii^	97.76 (9)	C25—C26—H26	119.4
C1—N1—Mg1	117.15 (12)	C27—C26—C25	121.3 (2)
C1—N1—C4	118.66 (15)	C27—C26—H26	119.4
C4—N1—Mg1	124.19 (12)	C26—C27—H27	119.8
N1—C1—C2	124.49 (18)	C26—C27—C28	120.39 (19)
N1—C1—C3	120.20 (17)	C28—C27—H27	119.8
C2—C1—C3	115.30 (17)	C27—C28—H28	119.6
C1—C2—C1^ii^	131.1 (2)	C27—C28—C29	120.7 (2)
C1^ii^—C2—H2	114.4	C29—C28—H28	119.6
C1—C2—H2	114.4	C24—C29—C33	121.59 (17)
C1—C3—H3A	109.5	C28—C29—C24	118.52 (19)
C1—C3—H3B	109.5	C28—C29—C33	119.86 (19)
C1—C3—H3C	109.5	C25—C30—H30	108.2
H3A—C3—H3B	109.5	C25—C30—C31	111.07 (19)
H3A—C3—H3C	109.5	C25—C30—C32	111.9 (2)
H3B—C3—H3C	109.5	C31—C30—H30	108.2
C5—C4—N1	118.02 (17)	C31—C30—C32	109.01 (19)
C9—C4—N1	121.63 (15)	C32—C30—H30	108.2
C9—C4—C5	120.33 (17)	C30—C31—H31A	109.5
C4—C5—C10	121.58 (19)	C30—C31—H31B	109.5
C6—C5—C4	118.5 (2)	C30—C31—H31C	109.5
C6—C5—C10	119.88 (19)	H31A—C31—H31B	109.5
C5—C6—H6	119.2	H31A—C31—H31C	109.5
C7—C6—C5	121.5 (2)	H31B—C31—H31C	109.5
C7—C6—H6	119.2	C30—C32—H32A	109.5
C6—C7—H7	120.1	C30—C32—H32B	109.5
C6—C7—C8	119.75 (19)	C30—C32—H32C	109.5
C8—C7—H7	120.1	H32A—C32—H32B	109.5
C7—C8—H8	119.2	H32A—C32—H32C	109.5
C7—C8—C9	121.6 (2)	H32B—C32—H32C	109.5
C9—C8—H8	119.2	C29—C33—H33	107.3
C4—C9—C13	123.19 (16)	C29—C33—C34	112.9 (2)
C8—C9—C4	118.29 (18)	C29—C33—C35	111.3 (2)
C8—C9—C13	118.51 (18)	C34—C33—H33	107.3
C5—C10—H10	107.6	C34—C33—C35	110.5 (2)
C5—C10—C11	110.4 (2)	C35—C33—H33	107.3
C5—C10—C12	112.8 (3)	C33—C34—H34A	109.5
C11—C10—H10	107.6	C33—C34—H34B	109.5
C11—C10—C12	110.6 (2)	C33—C34—H34C	109.5
C12—C10—H10	107.6	H34A—C34—H34B	109.5
C10—C11—H11A	109.5	H34A—C34—H34C	109.5
C10—C11—H11B	109.5	H34B—C34—H34C	109.5
C10—C11—H11C	109.5	C33—C35—H35A	109.5
H11A—C11—H11B	109.5	C33—C35—H35B	109.5
H11A—C11—H11C	109.5	C33—C35—H35C	109.5
H11B—C11—H11C	109.5	H35A—C35—H35B	109.5
C10—C12—H12A	109.5	H35A—C35—H35C	109.5
C10—C12—H12B	109.5	H35B—C35—H35C	109.5
C10—C12—H12C	109.5	C42—C41—C46	120.0
H12A—C12—H12B	109.5	C42—C41—C47	112.5 (10)
H12A—C12—H12C	109.5	C46—C41—C47	126.2 (10)
H12B—C12—H12C	109.5	C41—C42—H42	120.0
C9—C13—H13	108.4	C41—C42—C43	120.0
C9—C13—C14	111.60 (16)	C43—C42—H42	120.0
C9—C13—C15	110.67 (16)	C42—C43—H43	120.0
C14—C13—H13	108.4	C42—C43—C44	120.0
C15—C13—H13	108.4	C44—C43—H43	120.0
C15—C13—C14	109.19 (17)	C43—C44—H44	120.0
C13—C14—H14A	109.5	C45—C44—C43	120.0
C13—C14—H14B	109.5	C45—C44—H44	120.0
C13—C14—H14C	109.5	C44—C45—H45	120.0
H14A—C14—H14B	109.5	C44—C45—C46	120.0
H14A—C14—H14C	109.5	C46—C45—H45	120.0
H14B—C14—H14C	109.5	C41—C46—H46	120.0
C13—C15—H15A	109.5	C45—C46—C41	120.0
C13—C15—H15B	109.5	C45—C46—H46	120.0
C13—C15—H15C	109.5	C41—C47—H47A	109.5
H15A—C15—H15B	109.5	C41—C47—H47B	109.5
H15A—C15—H15C	109.5	C41—C47—H47C	109.5
H15B—C15—H15C	109.5	H47A—C47—H47B	109.5
Mg2—I2—Mg2^iii^	83.14 (3)	H47A—C47—H47C	109.5
I2—Mg2—I2^iii^	96.86 (3)	H47B—C47—H47C	109.5
I2—Mg2—Mg2^iii^	48.48 (2)	C56—C51—C52	132.0 (13)
I2^iii^—Mg2—Mg2^iii^	48.37 (2)	C56—C51—C57	120.2 (13)
N2^iv^—Mg2—I2	117.53 (5)	C57—C51—C52	107.6 (11)
N2—Mg2—I2^iii^	115.19 (5)	C51—C52—H52	127.6
N2^iv^—Mg2—I2^iii^	115.19 (5)	C53—C52—C51	104.8 (14)
N2—Mg2—I2	117.53 (5)	C53—C52—H52	127.6
N2—Mg2—Mg2^iii^	131.99 (5)	C52—C53—H53	118.7
N2^iv^—Mg2—Mg2^iii^	131.99 (5)	C54—C53—C52	122.6 (17)
N2^iv^—Mg2—N2	95.95 (9)	C54—C53—H53	118.7
C21—N2—Mg2	119.35 (12)	C53—C54—H54	112.5
C21—N2—C24	117.92 (15)	C53—C54—C55	135.0 (16)
C24—N2—Mg2	122.73 (12)	C55—C54—H54	112.5
N2—C21—C22	124.47 (18)	C54—C55—H55	127.3
N2—C21—C23	120.22 (17)	C56—C55—C54	105.4 (14)
C22—C21—C23	115.29 (18)	C56—C55—H55	127.3
C21^iv^—C22—C21	129.8 (2)	C51—C56—H56	120.0
C21^iv^—C22—H22	115.1	C55—C56—C51	120.1 (14)
C21—C22—H22	115.1	C55—C56—H56	120.0
C21—C23—H23A	109.5	C51—C57—H57A	109.5
C21—C23—H23B	109.5	C51—C57—H57B	109.5
C21—C23—H23C	109.5	C51—C57—H57C	109.5
H23A—C23—H23B	109.5	H57A—C57—H57B	109.5
H23A—C23—H23C	109.5	H57A—C57—H57C	109.5
H23B—C23—H23C	109.5	H57B—C57—H57C	109.5
			
Mg1—N1—C1—C2	10.2 (3)	N2—C24—C29—C33	−0.7 (3)
Mg1—N1—C1—C3	−171.06 (14)	C21—N2—C24—C25	−85.5 (2)
Mg1—N1—C4—C5	−82.4 (2)	C21—N2—C24—C29	96.2 (2)
Mg1—N1—C4—C9	95.97 (18)	C23—C21—C22—C21^iv^	−165.7 (2)
N1—C1—C2—C1^ii^	15.0 (5)	C24—N2—C21—C22	−169.8 (2)
N1—C4—C5—C6	179.90 (18)	C24—N2—C21—C23	8.7 (3)
N1—C4—C5—C10	−2.8 (3)	C24—C25—C26—C27	0.3 (3)
N1—C4—C9—C8	−179.70 (17)	C24—C25—C30—C31	−116.3 (2)
N1—C4—C9—C13	0.6 (3)	C24—C25—C30—C32	121.6 (2)
C1—N1—C4—C5	96.9 (2)	C24—C29—C33—C34	143.1 (2)
C1—N1—C4—C9	−84.7 (2)	C24—C29—C33—C35	−92.0 (2)
C3—C1—C2—C1^ii^	−163.7 (2)	C25—C24—C29—C28	3.0 (3)
C4—N1—C1—C2	−169.2 (2)	C25—C24—C29—C33	−178.97 (19)
C4—N1—C1—C3	9.5 (3)	C25—C26—C27—C28	1.5 (3)
C4—C5—C6—C7	−0.5 (3)	C26—C25—C30—C31	63.3 (3)
C4—C5—C10—C11	−95.4 (3)	C26—C25—C30—C32	−58.8 (3)
C4—C5—C10—C12	140.3 (2)	C26—C27—C28—C29	−1.0 (3)
C4—C9—C13—C14	125.58 (19)	C27—C28—C29—C24	−1.2 (3)
C4—C9—C13—C15	−112.6 (2)	C27—C28—C29—C33	−179.3 (2)
C5—C4—C9—C8	−1.3 (3)	C28—C29—C33—C34	−38.8 (3)
C5—C4—C9—C13	178.97 (18)	C28—C29—C33—C35	86.1 (3)
C5—C6—C7—C8	−0.6 (3)	C29—C24—C25—C26	−2.5 (3)
C6—C5—C10—C11	81.8 (3)	C29—C24—C25—C30	177.08 (18)
C6—C5—C10—C12	−42.4 (3)	C30—C25—C26—C27	−179.3 (2)
C6—C7—C8—C9	0.8 (3)	C41—C42—C43—C44	0.0
C7—C8—C9—C4	0.2 (3)	C42—C41—C46—C45	0.0
C7—C8—C9—C13	179.91 (19)	C42—C43—C44—C45	0.0
C8—C9—C13—C14	−54.1 (2)	C43—C44—C45—C46	0.0
C8—C9—C13—C15	67.7 (2)	C44—C45—C46—C41	0.0
C9—C4—C5—C6	1.5 (3)	C46—C41—C42—C43	0.0
C9—C4—C5—C10	178.7 (2)	C47—C41—C42—C43	−167.4 (8)
C10—C5—C6—C7	−177.8 (2)	C47—C41—C46—C45	165.6 (10)
Mg2—N2—C21—C22	10.1 (3)	C51—C52—C53—C54	−0.5 (14)
Mg2—N2—C21—C23	−171.40 (15)	C52—C51—C56—C55	2.8 (17)
Mg2—N2—C24—C25	94.59 (19)	C52—C53—C54—C55	4 (3)
Mg2—N2—C24—C29	−83.62 (19)	C53—C54—C55—C56	−4 (2)
N2—C21—C22—C21^iv^	12.9 (4)	C54—C55—C56—C51	0.4 (13)
N2—C24—C25—C26	179.31 (17)	C56—C51—C52—C53	−2.7 (14)
N2—C24—C25—C30	−1.1 (3)	C57—C51—C52—C53	−176.9 (10)
N2—C24—C29—C28	−178.82 (17)	C57—C51—C56—C55	176.4 (12)

Symmetry codes: (i) −*x*+1, −*y*+1, −*z*+1; (ii) *x*, −*y*+1, *z*; (iii) −*x*+1, −*y*+2, −*z*; (iv) *x*, −*y*+2, *z*.
